# High Levels of Stress Due to the SARS-CoV-2 Pandemic among Parents of Children with and without Chronic Conditions across the USA

**DOI:** 10.3390/children7100193

**Published:** 2020-10-21

**Authors:** Miranda A.L. van Tilburg, Emily Edlynn, Marina Maddaloni, Klaas van Kempen, Maria Díaz-González de Ferris, Jody Thomas

**Affiliations:** 1 Department of Clinical Research, College of Pharmacy and Health Sciences, Campbell University, Buies Creek, NC 27506, USA; mpmaddaloni0508@email.campbell.edu; 2 Division of Gastroenterology and Hepatology, Department of Medicine, University of North Carolina, Chapel Hill, NC 27514, USA; 3 School of Social Work, University of Washington, Seattle, WA 98105, USA; 4 Oak Park Behavioral Medicine, Oak Park, IL 60301, USA; eedlynn@opbmed.com; 5 Department of Economics, University of North Carolina at Chapel Hill (UNC), Chapel Hill, NC 27599, USA; klaasvk@live.unc.edu; 6 Division of General Pediatrics and Adolescent Medicine, Department of Pediatrics University of North Carolina, Chapel Hill, NC 27514, USA; maria_ferris@med.unc.edu; 7 Department of Psychiatry and Biobehavioral Sciences, Stanford University School of Medicine, Stanford, CA 94305-5101, USA; jody.thomas@megfoundationforpain.org

**Keywords:** SARS-CoV-2, COVID-19, parent stress, anxiety, depression, coping, resilience, self-efficacy, work stress, chronic diseases

## Abstract

Background: The 2020 SARS-CoV-2 pandemic led to community-wide measures affecting parents and children such as school/daycare closures, job losses, and interruptions in medical care for children with chronic diseases. This is the first study to describe the level of stress and mental health of parents of either healthy children or children with chronic conditions, during the 2020 pandemic. Methods: A representative sample of US parents was recruited from 10–17 April 2020. Parents completed online questionnaires about the past 7 days, including the Perceived Stress Scale, Resilient Coping Scale, Self-Efficacy Scale, Kansas Marital Satisfaction Scale, Parental Stress Scale, PROMIS Anxiety and Depression scales and various other pandemic-related stress questions Results: Levels of stressors (e.g., job loss, school closures, etc.) were high during this time (e.g., 79% of children attended home/online school) and parents reported being moderately to highly stressed. Rates of clinical anxiety (44.6%) and depression (42.2%) were high. Parents of children with chronic conditions reported higher levels of stress and worse mental health, but did not differ from other parents in dealing with stress or interruptions in work, child schooling, and marital satisfaction. Discussion: The COVID-19 pandemic has introduced unprecedented levels of stress for parents, especially those of children with chronic conditions. Mental health effects are expected to continue for months/years and preparation is needed to meet an increasing demand for mental health care.

## 1. Introduction

In normal times, many parents fulfill multiple roles at work as an employee and at home as a spouse and parent. When too many demands result from these multiple roles, expectations of each cannot be met and parents experience role overload [1]. Role overload has been associated with increased stress and negative outcomes at work as well as in the family [1]. Parents of children with chronic conditions are under particular stress as their parental role is often more demanding. Many studies have reported increased parenting stress among parents of children with chronic disorders [2,3].

Due to the unprecedented context of the 2020 pandemic caused by the SARS-CoV-2 pandemic, also called the COVID-19 pandemic, many parents have experienced increased role overload and stress as family life has been upended. In March 2020, several US states implemented orders to stay at home, except for essential work or shopping for essential needs. Most workplaces, schools and non-essential retail or services were closed, including temporarily halting non-emergency doctor visits [4]. Furthermore, people were asked to socially distance (keeping a distance of at least 6 ft between others) and quarantine at home when showing COVID-19 symptoms. The economic fall out of the stay at home orders was large, with 43% of Americans in April 2020 reporting to have lost a job or taken a pay cut [5]. One report found about half of all people in the US worked from home early in the pandemic [6]. Furthermore, according to the National Center for Education Statistics, school closures affected 55.1 million students across the US in April 2020 [7], with most schools switching to online learning. Stay at home orders and extra protection of the elderly has also isolated families from much of their support system and childcare options, with daycare centers, sitters and grandparents generally unavailable to help parents. In addition, parents of children with chronic conditions may have an additional loss of support and services from the medical community because of the closures of health care facilities and the move to virtual clinical encounters.

In a call to action for mental health science, 24 world experts indicated that an “immediate priority is collecting high-quality data on the mental health effects of the COVID-19 pandemic across the whole population and vulnerable groups.” [8]. Data have been published showing global rates of psychological distress that are worrisomely high, with some studies finding a doubling of clinical anxiety and depression among adults [9]. Parents particularly are negatively affected, with one study reporting that almost one out of three parents reported a worsening of their mental health during the pandemic [10]. It seems reasonable that parents of children with chronic diseases are hardest hit by the many stressors created due to the pandemic, such as loss of childcare, loss of services through school and loss of access to medical care. This is the first study to describe and compare the level of stress and mental health of parents of either healthy children or children with chronic conditions, during the 2020 pandemic.

## 2. Materials and Methods

### 2.1. Sample

A representative sample of the US population was recruited through CINT USA, Inc. (Atlanta, GA, USA, www.cint.com). CINT offers access to more than 19 million worldwide individuals, interested in completing surveys. We targeted our recruitment to include 300 parents of healthy children (ages 0–18) and 300 parents of children with chronic conditions. We targeted parents of children with 43 different diseases such as anxiety, Attention deficit hyperactivity disorder (ADHD), cancer and diabetes. As of the date of data collection, no evidence was available indicating that any childhood disease increases the risk of severe COVID-19 illness [11]. Furthermore, as described in the introduction, taking care of a child with a chronic illness increases parent stress and the current study was mostly focused on stress in parents during the 2020 pandemic.

Sampling was targeted to include parents (no more than 60% women) across all 50 states, and be consistent with ethnic (18% Hispanic), race (76% white, 13% African American) and socioeconomic status (15% no high school degree) make-up of the US population per censuses from 2010–2018 (retrieved from: https://wonder.cdc.gov/). Recruitment was completed between 10 and 17 April 2020. All subjects gave their informed consent for inclusion before they participated in the study. The study was conducted in accordance with the Declaration of Helsinki, and the protocol was approved by the Campbell University Institutional Review Board (#586).

### 2.2. Measures

After screening and electronic consent, parents completed a questionnaire on stress related to the SARS-CoV-2 pandemic lockdowns. The questions were organized into subsections based on Bronfenbrenner’s ecological systems theory [12], which divides influences on stress into the following layers:

(1) Intrapersonal:
  - Perceived Stress was measured by the 10-item Perceived Stress Scale, which has been widely validated and is highly reliable [13]. Cronbach’s alpha in the current sample was 0.83. An example item is: “In the last week, how often have you been upset because of something that happened unexpectedly?” scored on a 5-point Likert scale ranging from “never” (1) to “very often” (5). All items are summed to create a total score.
  - Coping was measured by the Brief Resilient Coping Scale [14] as well as a COVID-19 self-efficacy scale. The Brief Resilient Coping scale contains four items scored on a 5-point Likert scale ranging from “does not describe me at all” to “describes me very well”. An example item is: “Please indicate how you have been dealing with stress in the past 7 days—I actively look for ways to replace the losses I encounter in life”. Internal reliability was high (0.83) in the current study. The COVID-19 self-efficacy scale was developed for this study based on guidelines by Bandura [15]. Cronbach’s alpha (0.93) for this scale was high in the current study, indicating good internal reliability. COVID-19 self-efficacy also correlated with anxiety (r = −0.24), coping (r = 0.41), and perceived stress (r = −0.38), indicating validity. An example item is: “Rate your level of confidence about your current ability to do these tasks:—Keep my family physically safe from the virus” rated on an 0–10 scale ranging from “cannot do at all” to “highly certain I can do”. All scores are summed to create a total score.
(2) Microsystem/Family:
  - Marital satisfaction was measured with the Kansas Marital Satisfaction Scale, a validated and reliable measure [16] (three questions scored on a 7-point Likert scale). Internal reliability in our sample was high (α = 0.96). An example item is: “In the past 7 days, how satisfied have you been with your marriage/relationship?” Items are summed to create a total score.
  - Parenting Stress was measured by the Parental Stress Scale [17] (18 items scored on a 5-point Likert scale). Previous studies have shown this scale is highly reliable [17]. Cronbach’s alpha for this scale in the current study was reasonable (0.78). An example item is: “In the past 7 days, it is difficult to balance different responsibilities because of my child(ren).”, answered on 5-point scale ranging from “strongly disagree” to “strongly agree”. All items are summed to create a total score.
(3) Mesosystem:
  - Social support was assessed by indicating who is available to help with childcare besides the parent, as well as how much a spouse/partner is available for contributing to childcare, work and chores (rated on a 5-point Likert scale ranging from “I do most” to “partner does most”).
  - Work stress was measured by asking about changes in job status (job loss, reduced hours/pay, working from home), and a rating of job stress (on a 10-point scale). Items included changes in child’s schooling as well as a rating of parent stress related to online schooling (if applicable, rated on 10-point scale).
(4) Exosystem:
  - The degree of restrictions in the community and worries around infection as well as exposure to SARS-CoV-2 infection were assessed.

In addition to stress, mental health was assessed. Anxiety and depression were measured by the PROMIS six-item short-form scales, which have excellent reliability and validity [18]. Internal reliability of these scales in the current study was high (0.92 and 0.93). An example item is: “In the past 7 days, have you felt—Nervous” scored on a 5-point scale ranging from “never” to “always”. Scores are summed, and the raw score converted to a T-score (per existing guidelines). Norm scores are provided, with >55 considered mild to severe anxiety/depression [18]. Frequency of physical symptoms, often associated with anxiety or depression, such as pain, eating and sleeping problems, was assessed in the number of days in the past week.

All questionnaires/questions asked parents to report about the past 7 days.

### 2.3. Data Analysis

Description of stressors, coping, perceived stress and mental health are depicted as means + standard deviations for continuous variables, and frequency + percentages for categorical variables. Stressors, perceived stress coping and mental health were compared between parents of healthy children, parents of children with physical conditions and parents of children with mental health conditions using one-way ANOVA for continuous variables (with Tukey’s post hoc tests) and chi-squared tests for categorical variables (with additional chi-squared tests to test for group differences, if the overall test was significant). To adjust for type 1 errors due to multiple testing, a *p* level of 0.01 was chosen to be significant.

## 3. Results

### 3.1. Demographics

A total of 628 parents completed the survey. We removed six subjects who repeated the questionnaire as well as 12 subjects who had inconsistent answers on two quality assurance questions (parent age and number of children) to determine the consistency of responding. As can be seen in Table 1, the final sample was representative of the quotas/US population according to gender, race and ethnicity. Parents were from all 50 states in the US, with more participants from populous states. For example, 9.7% (*n* = 59) of participants lived in California and 0.5% (*n* = 3) in Delaware. By design, half of the sample was parents whose children had chronic conditions, such as asthma, autism, attention deficit hyperactivity disorder, anxiety and diabetes (see Table 1, depicting the most frequent diagnoses). No differences were found in demographic variables between parents of children with and without chronic conditions, except for child age. A total of 158 children had physical health disorders and 134 had mental health disorders (10 reported both and were included in the “physical health disorder” group for the purpose of our analyses). The youngest child was slightly older in the disease group versus the no disease group.

### 3.2. Pandemic Stressors

Community lockdowns affected almost all households: Social distancing (*n* = 573, 93.9%), self-isolation (*n* = 326, 53.4%), shelter in place (*n* = 354, 58.0%) and quarantine (*n* = 188, 30.8%) were common community restrictions. In addition, 14.4% of parents and 13.8% of children were reported to have had SARS-CoV-2 infection (by test or suspected). Children with physical chronic conditions were more likely to have (suspected) SARS-CoV-2 infection than those with mental health conditions or without chronic conditions (see Table 2). Furthermore, significant differences were found between all three groups in the percentage of parents rating their children at high risk of becoming sick (46.8% physical health conditions, 34.4% mental health conditions, 7.8% no chronic conditions), and reported their child to be at high risk of becoming very sick with COVID-19.

### 3.3. Stressors, Perceived Stress and Coping

This sample of parents showed overall stress levels, as measured on the perceived stress scale, in the moderate range, with parents of children with chronic conditions reporting more stress than those without chronic conditions (see Table 2). Pandemic-related stress (measured on a 0–10 scale) was higher in parents of children with chronic physical conditions compared to parents of children with mental health conditions or parents of healthy children (see Table 2). Parents in the chronic condition group reported their child’s illness was affected by the pandemic “somewhat” or “to a great extent” (59.6%). This number did not differ significantly between those with mental health (58.2%) vs. physical health conditions (65.2%, *p* = 0.052). Resilient coping and COVID-19 self-efficacy were not different between parents of children with and without chronic conditions (see Table 2).

### 3.4. Parenting-Related Stressors and Perceived Stress

Thirty-nine percent of parents reported that dealing with children is more stressful than before pandemic restrictions and this was not different across the three groups of parents (see Table 2). Most children were homeschooled or in online school and this was found to be moderately stressful. Many parents (65.5%) could rely on their spouse/partner for help. Since women spend more time on housework/childcare than men [19], we examined how much women contributed versus men during the 2020 pandemic. Women reported contributing more than their partner towards childcare (61.2% women vs. 37% men, *p* < 0.001) and household chores (67.0% vs. 39.4%, *p* < 0.001). Other forms of childcare, such as grandparents (15.1%), other family members (10.3%), sitters (1.1%) and daycare (1%), were low. Almost one in four parents (22.6%) had no one to rely on for childcare in the past week. This level of support did not statistically differ between parents of children with and without chronic conditions. Marital satisfaction appeared to be moderately high and not different between the three parent groups (see Table 2).

### 3.5. Work-Related Stressors and Perceived Stress

Job loss or reduced pay/hours were common (see Table 2), affecting about half of parents. Forty-two percent of parents transitioned to working from home, and one in three was considered an essential worker (see Table 2). Approximately 1/3 of parents (37.6%, n = 227) worried about the future of their jobs “a lot” or “a great deal” and 30% of parents found work “always” or “often” stressful in the past 7 days.

### 3.6. Mental Health

Parents reported mild to moderate levels of depression and anxiety (See Table 2). Stress-related physical symptoms occurred on average 2–3 days a week (see Table 2). These reactions to stress were higher in the parents of children with chronic conditions than those without chronic conditions. Stomachaches were different between all three groups, with parents of children with physical conditions reporting the most, and parents of healthy children reporting the least stomachaches.

## 4. Discussion

The mental health effects of the 2020 SARS-CoV-2 pandemic are yet to be fully understood. In this nationally representative study, we found that levels of stress among parents were moderate to high but additionally so for parents of children with either physical or mental health chronic conditions. Stressors due to community restrictions during the pandemic were high: the vast majority of families practiced social distancing, over half of parents lost their job or had reduced income, 42% transitioned to working from home and three out of four children were schooled at home/online. In the meanwhile, traditional support systems for childcare dwindled, with 1% of parents having access to sitters or daycare and 15% able to rely on grandparents (compared to 38% in a 2018 AARP survey [20]). The reduction in childcare by grandparents is likely not only an effect of social distancing but also to protect the elderly from SARS-CoV-2 infection and COVID-19. In a study conducted in June (two months after our study) 24% of parents reported losing care during the pandemic [10], supporting our findings. Many of these stressors add to the already moderately high stress levels in Americans, which in 2019 were reported on a scale of 1–10 with an average of 4.9 in the past month [21]. The stress level in our sample was 6.7 in the past 7 days. In a non-peer reviewed study, by the American Psychological Association, similar stress levels were found among parents during the 2020 pandemic, while adults without children were significantly less stressed (average stress level 6.7 vs. 5.5 in the past month) [22]. Thus, during the 2020 pandemic, parents report a high level of stress.

Rates of clinical anxiety (44.6%) and depression (42.2%; based on norm scores) were high among parents, and substantially above rates from previous studies. For example, based on the same PROMIS norm scores, the rates of depression (10%) and anxiety (17%) in adults with a musculoskeletal condition were much lower than the rates found in the current sample [23]. Furthermore, a meta-analysis of studies in conflict areas, a high-stress environment, found 22% of adults to be clinically depressed or anxious [24]. Even during the COVID-19 outbreak in China, rates of depression (20.1%) and anxiety (35.1%) were lower in adults compared to our sample of parents [25]. Although no statistical test can be performed to find if these differences before and during the pandemic are statistically significant, they seem to be clinically significant. These data suggest that anxiety and depression rates for parents during the SARS-CoV-2 pandemic were twice as high as in these comparable studies. Clearly, our results support that during the SARS-CoV-2 pandemic, mental health of parents was suboptimal.

Not surprisingly, stress levels, anxiety and depression were significantly higher in parents who take care of children with chronic conditions (physical or mental) compared to parents of healthy children. Not only is the care of a child with a chronic condition stressful in itself, the effects of the pandemic restrictions also reduced social support for childcare, increased financial concern due to loss of income and introduced the new worry about COVID-19 health effects. This is simultaneous with reduced access to medical care and increased stress when receiving care because of infection concerns. A recent study found that the pandemic resulted in delays in medical care for 39% of children [10]. The impact of these stressors is greater for those caring for a child with a chronic condition. Our data suggest that parents of children with chronic conditions are a group that needs additional support and intervention while SARS-CoV-2 and related community restrictions continue as a threat.

This study has many strengths: it is the first to describe parenting stress during times of the SARS-CoV-2 pandemic and is based on a representative sample of the USA. We examined specifically the effects on parents of children with a variety of chronic conditions, including common mental and physical health disorders. The main limitation of our study is the cross-sectional design, capturing only one time point during the 2020 pandemic. Longitudinal data are needed. In addition, the study is descriptive and includes no direct comparisons with pre-pandemic levels. Third, all data are collected by self-report. Childhood chronic conditions included in the study varied widely from ADHD to cancer. We chose this to provide a general overview of the level of stress during the pandemic of parents who have a child with a chronic disorder. However, future studies are needed to examine levels of parenting stress during the 2020 pandemic by disorder as the impact may vary for each. Lastly, the sample was recruited through a commercially available recruitment service. These parents volunteer to be contacted for survey research and this may introduce bias in our sampling. However, we and others have previously used the same sampling methods for nationally representative samples [26,27].

## 5. Conclusions

In sum, the 2020 SARS-CoV-2 pandemic has introduced unprecedented levels of stress for parents, especially those of children with chronic conditions. More data are needed to look at the impact on children, but it is not difficult to imagine it being substantial, as parental mental health significantly impacts the child’s experience. These findings emphasize the need to support parents while pandemic community restrictions continue to significantly interfere with daily life, and find ways to help families deal with the aftermath of this collective experience. Mental health effects of this pandemic for children and parents are expected to continue for months or years. Studies like this one offer a description of the problem; health care preparation needs to follow to identify targeted interventions tailored to the specific challenges of the pandemic experience. Innovation will be needed to do that on a large scale to meet this increased demand in mental health care.

## Figures and Tables

**Table 1:** Sample demographics.

	Parents of Healthy Children	Parents of Children with Chronic Conditions	*p*-Value *
	N = 308	N = 302	
	Mean (SD) or Frequency (%)	Mean (SD) or Frequency (%)	
Age	40.61 (10.6)	40.61 (9.7)	0.998
Race			0.021
Caucasian	208 (67.5%)	232 (76.8%)	
African American	51 (16.6%)	47 (15.6%)	
Hispanic	46 (14.9%)	50 (16.6%)	0.582
Median income	USD 50.000–75.000	USD 50.000–75.000	0.105
Number of children	1.74 (1.0)	1.90 (1.0)	0.064
Age youngest child	8.30 (5.2)	9.47 (4.9)	0.005
Private insurance	195 (63.7%)	178 (59.5%)	0.190
Chronic health condition (At least *n* = 10 children; other conditions can be requested from authors)	N/A		
Asthma		111 (18.2%)	
Autism		37 (6.1%)	
Attention deficit		50 (8.2%)	
disorder			
Anxiety		32 (5.2%)	
Diabetes		22 (3.5%)	

N = number of participants * To adjust for type 1 errors due to multiple testing, a *p* level of 0.01 was chosen to be significant.

**Table 2:** Pandemic-related stressors, perceived stress, coping and mental health among parents of healthy children or children with chronic conditions.

	Parents of Healthy Children	Parents of Children with Physical Conditions	Parents of Children with Mental Conditions	*p*-Value *
	N = 308	N = 168	N = 134	
	Mean (SD) or N (%)	Mean (SD) or N (%)	Mean (SD) or N (%)	
Parent SARS-CoV-2				
Confirmed by test	12 (3.9%)	23 (13.7%)	7 (5.2%)	<0.001
Suspected	15 (4.9%)	25 (14.9%)	6 (4.5%)	<0.001
Child SARS-CoV-2				
Confirmed by test	11 (3.6%)	23 (13.7%)	8 (6.0%)	<0.001
Suspected	14 (4.5%)	22 (13.1%)	6 (4.5%)	<0.001
Work Stressors due to Pandemic				
Lost job	43 (14%)	35 (20.8%)	16 (11.9%)	0.063
Reduced pay	118 (38.3%)	77 (45.8%)	56 (41.8%)	0.277
Work from home	121 (39.3%)	74 (44.0%)	61 (45.5%)	0.386
Essential worker	94 (30.5%)	55 (32.7%)	57 (42.5%)	0.046
Parenting Stressors/Stress				
Children switched to home/online school	221 (72.70%)	148 (88.63%)	114 (85.07%)	<0.001
How stressful is online schooling?	5.25 (2.9)	5.86 (3.0)	5.75 (2.6)	0.099
1–10 scale				
Parenting more stressful due to pandemic	135 (44.9%)	68 (40.7%)	67 (50.0%)	0.014
Kansas Marital Satisfaction Scale	6.34 (4.7)	6.07 (3.7)	6.34 (4.2)	0.825
General Stress				
Perceived Stress Scale	16.41 (7.1) moderate **	18.69 (5.5) moderate **	17.99 (7.7) moderate **	0.001
Stress related to pandemic	6.25 (2.6)	7.49 (2.3)	6.63 (2.6)	<0.001
1–10 scale				
Dealing with Stress				
COVID self-efficacy	69.64 (15.8)	66.92 (16.7)	65.94 (16.0)	0.049
Resilient coping	15.2 (2.9)	15.05 (3.1)	14.42 (3.4)	0.065
Mental Health				
PROMIS depression (T-score)	52.52 (13.1) none **	59.33 (13.0) mild **	56.16 (13.0) mild **	<0.001
PROMIS anxiety (T-score)	55.8 (10.7) mild **	61.31 (10.6) moderate **	59.09 (10.8) mild **	<0.001
Stomachaches (#days/week)	1.45 (1.2)	2.77 (2.0)	2.01 (1.5)	<0.001
Headaches (#days/week)	1.93 (1.3)	3.17 (2.7)	3.01 (1.9)	<0.001
Sleep problems (#days/week)	2.13 (1.9)	3.45 (2.4)	3.59 (2.4)	<0.001
Eating problems (#days/week)	1.48 (1.4)	2.57 (2.1)	2.24 (2.1)	<0.001

* To adjust for type 1 errors due to multiple testing, a *p* level of 0.01 was chosen to be significant. ** Based on published norm scores.
